# Editorial: Precision nutrition and nutrients: making the promise a reality

**DOI:** 10.3389/fnut.2025.1553149

**Published:** 2025-01-27

**Authors:** Sunmin Park

**Affiliations:** ^1^Department of Bioconvergence, Hoseo University, Asan, Republic of Korea; ^2^Department of Food and Nutrition, Obesity/Diabetes Research Center, Hoseo University, Asan, Republic of Korea

**Keywords:** precision nutrition, genetic variants, amino acid bioavailability, gene-lifestyle interaction, ketogenic diet

## 1 Introduction

Precision nutrition offers a transformative approach that transcends generic dietary guidelines, providing individualized strategies based on genetic, metabolic, and environmental variability (1). Unlike traditional nutrition frameworks, precision nutrition optimizes health outcomes by addressing specific genetic variations of individuals that influence nutrient metabolism, such as single-nucleotide polymorphisms (SNPs) linked to differential responses to vitamins, lipids, and other nutrients to influence metabolic disease risk (2–4).

In addition to genetics, the gut microbiome modulates the diet-health relationship (5, 6). Although not directly addressed in this Research Topic, the microbiome's importance in nutrient absorption and metabolism is undeniable (7, 8). Genetic predisposition interacts with lifestyle behaviors including stress, sleep, physical activity, dietary intake, and environmental exposures, emphasizing the need for integrative frameworks in precision nutrition (9, 10).

This Research Topic features five studies exploring genetic and methodological advancements in precision nutrition, including SNPs influencing carotenoid bioavailability, gene-lifestyle interactions in lipoprotein metabolism, and machine-learning applications in predicting glycemic responses. Collectively, these contributions demonstrate the growing potential of precision nutrition for disease prevention and public health strategies.

## 2 Overview of the Research Topic

The featured studies encompass diverse approaches to advancing precision nutrition (Von Holle et al.; Paoletti et al.; Liu et al.; Park et al.; Hur et al.).

One study included in this Research Topic substantially expands the understanding of novel SNPs (Von Holle et al.) in the regulation of carotenoids, particularly lutein, and zeaxanthin. It also includes a comprehensive review of genome-based personalized nutrition technologies (Park et al.). Other studies address amino acid bioavailability and gene-lifestyle interactions in lipoprotein metabolism (Hur et al.; Paoletti et al.; Liu et al.), collectively highlighting the role of genetic variations in tailoring dietary interventions. These findings form a robust framework for translating precision nutrition research into actionable clinical applications.

## 3 Theme integration

### 3.1 Genetic basis of precision nutrition

Advances in understanding the genetic factors influencing nutrient metabolism have laid the foundation for precision nutrition. Previous studies have shown that polymorphisms in the *BCO1* gene are associated with the metabolism of carotenoids, particularly beta-carotene (11). The current investigation, however, identifies novel SNPs (rs6564851-C and rs6420424-A) located upstream of the *BCO1* gene, providing new insights into the genetic regulation of carotenoid metabolism (Von Holle et al.). These specific SNPs were found to significantly impact circulating levels of lutein and zeaxanthin, further elucidating how genetic variations influence the nutrient responses of individuals (Von Holle et al.). Additionally, the patent review in this Research Topic highlights the expanding importance of genome-based personalized nutrition technologies (Park et al.). It explores how genetic information is increasingly integrated into personalized dietary recommendations, particularly those tailored to disease prevention and management (Park et al.). While earlier reviews primarily focused on genetic insights without emphasizing their application (12), this study emphasizes the translation of genetic discoveries into commercial and clinical settings, illustrating the practical implementation of precision nutrition approaches. This study advances the field by describing how genetic discoveries are translated into clinical and commercial applications.

### 3.2 Dietary interventions and nutrient bioavailability

The investigation into amino acid bioavailability, using the indicator amino acid oxidation method, builds on earlier research that focused primarily on static measures of amino acid content to assess protein quality (13). The metabolic availability assessment of amino acids provides a more refined understanding of protein quality by accounting for factors beyond simple digestibility, particularly for amino acids susceptible to heat and processing modifications (Paoletti et al.). Furthermore, the exploration of ketogenic therapy in treating brain diseases represents an expansion of precision nutrition approaches, demonstrating how specific dietary interventions can be tailored for therapeutic outcomes. This therapeutic strategy shows promise across various neurological conditions, with emerging evidence supporting its efficacy through altered patient metabolism and ketone production (Liu et al.). The novelty of these findings lies in the adaptation of specific dietary interventions to optimize therapeutic outcomes through precise metabolic assessment and dietary modification. Together, these studies illustrate the growing promise of precision nutrition to improve health outcomes through integrating cutting-edge methodologies in nutrient bioavailability assessment and personalized dietary interventions.

### 3.3 Lifestyle and polygenic interactions

The critical role of individual genetic variants, such as *CETP_rs708272*, in modulating HDL-C levels and cardiovascular risk is well established (14, 15). However, this Research Topic presents new research identifying additional SNPs, including *ZPR1_rs3741297, BUD13_rs180327*, and *ALDH1A2_rs588136*, that interact with environmental factors, such as dietary energy intake and sulfur-containing microbial diets, that modulate HDL-C levels (Hur et al.) in contrast to previous studies that predominantly focused on the effects of single-gene variants (16). This research introduces a more elegant and comprehensive approach by interjecting multiple genetic variants and their interactions with lifestyle factors. This multifactorial perspective is novel in its application to precision nutrition, highlighting the importance of integrating genetic predisposition with environmental influences to optimize dietary interventions. It advances a significant advance in the understanding of gene-environment interactions, paving the way for more personalized dietary recommendations aimed at improving lipoprotein metabolism and overall health.

## 4 Path forward: precision nutrition and genetic predispositions

**Figure 1:**
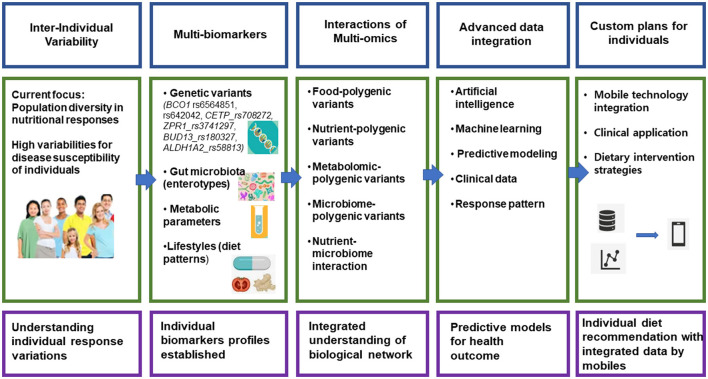
Framework for implementation of precision nutrition. This image illustrates a comprehensive framework for developing personalized nutrition recommendations, flowing from left to right across five key stages. It begins with understanding individual variability in how people respond to nutrition, then moves through collecting multiple biomarkers (including genetic variants and gut microbiota), analyzing various “-omics” interactions (like food-polygenic variants), using advanced data integration with artificial intelligence and machine learning, and finally delivering customized nutrition plans through mobile technology. The bottom row provides a concise summary of each stage's goal, ultimately leading to personalized diet recommendations delivered via mobile devices.

The future of precision nutrition hinges on integrating multi-omics data with advanced computational tools, as illustrated in Figure 1. While genome-wide association studies continue to reveal genetic variants affecting nutrient metabolism, metabolomic profiling enhances our understanding of individual responses (7). Machine learning algorithms have proven valuable in predicting metabolic responses, facilitating the transition from theoretical research to practical applications.

Translating genetic discoveries into actionable dietary recommendations remains crucial. While polygenic risk scores combined with lifestyle factors provide a framework for personalized dietary guidance (17), current research is limited by its predominant focus on European populations (18, 19). Beyond expanding population diversity, emerging evidence highlights specific genetic variants, such as missense mutations and those in UTR regions, as potential targets for nutrient-based interventions, offering innovative approaches to disease prevention and management (4, 20).

Recent technological advances, particularly in mobile applications, are democratizing access to precision nutrition tools. These developments, combined with emerging systems biology approaches integrating microbiome, proteomics, and metabolomics data, promise more nuanced dietary interventions (21, 22). This comprehensive approach will enable the development of more effective, personalized nutrition strategies suitable for clinical implementation.

## 5 Conclusion

The studies in this Research Topic demonstrate significant advances in precision nutrition, from genetic variant identification to dietary optimization. While challenges remain in integrating multi-omics data and scaling personalized approaches, these findings provide a clear path forward. The potential to optimize health outcomes through interventions tailored to genetic, metabolic, and lifestyle factors represents a promising direction toward personalized nutritional guidance that considers individual requirements while promoting broader public health.
